# The emerging genomics and systems biology research lead to systems genomics studies

**DOI:** 10.1186/1471-2164-15-S11-I1

**Published:** 2014-12-16

**Authors:** Mary Qu Yang, Kenji Yoshigoe, William Yang, Weida Tong, Xiang Qin, A Keith Dunker, Zhongxue Chen, Hamid R Arbania, Jun S Liu, Andrzej Niemierko, Jack Y Yang

**Affiliations:** 1 MidSouth Bioinformatics Center, Department of Information Science, George W. Donaghey College of Engineering and Information Technology, University of Arkansas at Little Rock, 2801 S. University Avenue, Little Rock, Arkansas, 72204, USA; 2 Joint Bioinformatics Graduate Program, University of Arkansas at Little Rock and University of Arkansas for Medical Sciences, Little Rock, Arkansas 72204, USA; 3 Department of Computer Science, George W. Donaghey College of Engineering and Information Technology, University of Arkansas at Little Rock, 2801 S. University Ave, Little Rock, Arkansas 72204, USA; 4 Division of Bioinformatics and Biostatistics, National Center for Toxicological Research, Unites States Foods and Drug Administration, 3900 NCTR Road, Jefferson, Arkansas 72079, USA; 5 Human Genome Sequencing Center, and Department of Molecular and Human Genetics, Baylor College of Medicine, Houston, Texas 77030, USA; 6 Center for Computational Biology and Bioinformatics, Indiana University School of Medicine, Indianapolis, Indiana 46202, USA; 7 Department of Epidemiology and Biostatistics, Indiana University School of Public Health, 1025 E. 7th Street, PH C104, Bloomington, Indiana 47405, USA; 8 Department of Computer Science, University of Georgia, Athens, Georgia 30602, USA; 9 Department of Statistics, Harvard University, Cambridge, Massachusetts 02138, USA; 10 Division of Biostatistics and Biomathematics, Department of Radiation Oncology, Massachusetts General Hospital and Harvard Medical School, Boston, Massachusetts 02114, USA

## Abstract

Synergistically integrating multi-layer genomic data at systems level not only can lead to deeper insights into the molecular mechanisms related to disease initiation and progression, but also can guide pathway-based biomarker and drug target identification. With the advent of high-throughput next-generation sequencing technologies, sequencing both DNA and RNA has generated multi-layer genomic data that can provide DNA polymorphism, non-coding RNA, messenger RNA, gene expression, isoform and alternative splicing information. Systems biology on the other hand studies complex biological systems, particularly systematic study of complex molecular interactions within specific cells or organisms. Genomics and molecular systems biology can be merged into the study of genomic profiles and implicated biological functions at cellular or organism level. The prospectively emerging field can be referred to as systems genomics or genomic systems biology.

The Mid-South Bioinformatics Centre (MBC) and Joint Bioinformatics Ph.D. Program of University of Arkansas at Little Rock and University of Arkansas for Medical Sciences are particularly interested in promoting education and research advancement in this prospectively emerging field. Based on past investigations and research outcomes, MBC is further utilizing differential gene and isoform/exon expression from RNA-seq and co-regulation from the ChiP-seq specific for different phenotypes in combination with protein-protein interactions, and protein-DNA interactions to construct high-level gene networks for an integrative genome-phoneme investigation at systems biology level.

## Introductory review

Synergistic integrating multi-layer genomic data at a systems level can provide deeper insights into the molecular mechanisms related to disease initiation and progression, and also guide many pathway-based biomarker and drug target identifications.

Traditionally genomics is to study sequence, structure, and function of a genome. With the advent of high-throughput next-generation sequencing technologies, sequencing RNAs from specific cells or organisms provide much useful information that can include non-coding RNAs, novel RNAs, direct measurement of RNA sequences, gene expression, differential isoforms and alternative splicing. Systems biology is related to genomics in the study of complex biological systems particularly systematic study of complex molecular interactions within cells or organisms. Genomics and systems biology significantly overlap and interact in the aspect of utilizing genomic information and the implicated biological functions at cellular or organism level. These two disciplines merge at the cross field that can be referred as systems genomics or genomic systems biology.

Systems biology approaches can help genomics studies through systematic and integrative approaches to incorporate multiple genomic data and assemble the information toward cellular or physiological behaviours related to a particular biological phenotype such as disease. While gene expression is organ, tissue or cellular specific, genomic mutations can infer disease causal alterations, and ChIP-seq can provide co-regulation information, henceforth multi-layer genomic data can be integrated to reveal the mechanisms of diseases. Genomics and molecular systems biology research overlap and could be merged into a field that can be referred to as systems genomics. As many types of diseases are resulted from multiple genetic alterations, the prospective systems genomics research can provide a blueprint to pinpoint the disease associated mutations from the study of individual genomes, biological pathways and interaction networks. Bearing this in mind, the Mid-South Bioinformatics Centre (MBC) is particularly interested in promoting education and research advancement in this prospectively emerging field. Based on the past investigations and research outcomes, MBC is further utilizing differential gene and isoform/exon expression from RNA-seq and co-regulation derived from ChIP-seq data specific for different phenotypes in combination with protein-protein and protein-DNA interactions to construct high-level gene networks for an integrative genome-phoneme investigation at systems biology level. Such investigations involve further developments of computational intelligence and molecular biology techniques toward the identification of biomarkers from the gene networks for early disease diagnosis and effective drug target identification. The research leverages genomic mutation information including whole exome SNPs (single-nucleotide polymorphisms) with network-based genome-wide association analysis for identifying disease-related genes and networks at systems level. Furthermore, combining with other approaches, the research can also lead to identify genome structural variations such as copy number variations and further functional analysis on the proteins and pathways in these networks to unravel complex mechanisms of disease initiation and potential drug targets.

Given above objectives, MBC has restructured its research themes and presented new research initiatives at the 2014 International Conference on Advances in Big Data Analytics to promote the computational big data research in translational bioinformatics and genomics. The conference received hundreds of research papers worldwide and the International Society of Intelligent Biological Medicine (ISIBM) provided academic sponsorship to the 2014 international conference. Each paper was peer reviewed by the conference program committee members http://www.world-academy-of-science.org/worldcomp14/ws/conferences/abda14/committee and external reviewers. ISIBM decided to form a dedicated review committee chaired by Dr. A. Keith Dunker, President of ISIBM, Founding Director and T. K. Li Professor for Medical Research at Indiana University School of Medicine Centre for Computational Biology and Bioinformatics to select high-quality papers based on peer-reviews for this special *BMC Genomics* supplement. ISIBM Vice President Dr. Dong Xu, James C. Dowell Professor and Chair of Computer Science Department of University of Missouri and Vice-President Dr. Hamid R. Arabnia, Professor of Computer Science at University of Georgia served on the review committee. In addition, ISIBM Secretary-General Dr. Yunlong Liu, Associate Editor of *BMC Genomics*, Associate Professor of Molecular and Medical Genetics and Director of Bioinformatics Core at Indiana University School of Medicine, Indiana University Purdue University Indianapolis, Dr. Zhongxue Chen, Associate Editor of *BMC Genomics* and Director of Study Design and Data Analysis Consulting Centre at Indiana University Bloomington, Dr. Xiang Qin, Assistant Professor of Molecular and Human Genetics at the Human Genome Sequencing Centre of Baylor College of Medicine, along with Dr. Weida Tong, Director of HHS/FDA/NCTR Division of Bioinformatics, and Biostatistics and Professor of Bioinformatics, University of Arkansas at Little Rock (joint bioinformatics Ph.D. program core faculty by courtesy), and Dr. Youping Deng, Associate Editor of *BMC Research Notes*, Associate Professor of Medicine and Director of Cancer Bioinformatics and Biostatistics at Rush University Medical Centre at Chicago also joined the committee. The committee invited external experts in the fields to review all submitted papers and selected 4 significant papers based on peer reviews for this special *BMC Genomics* supplement.

In this *BMC Genomics *supplement, Deng's laboratory and collaborators presented integrative experimental and computational genomics approaches at systems level to study the impact of RDX induced toxicity using rats as model organism [1]. The investigators extracted total RNA from both RDX exposed group and control group. They developed pipeline methods from microRNA (miRNA) and messenger RNA (mRNA) profiling to differential gene expression, pathway and network analysis. The regulatory roles of miRNAs on mRNAs were investigated systematically. Pathway and network analyses were performed to study significantly regulated genes. Such comprehensive experimental and computational investigation at systems biology level can generate a significant impact beyond the studying subject of RDX itself, as the paper provides a model of systematic approaches that can be applied to many studies using integrative genomics and systems biology techniques.

PDB (RCSB Protein Data Bank) contains not only redundant but also some poor quality data. Xu’s laboratory developed MUFOLD-DB, a web-based database, to automatically collect and process the PDB files, thereby provides users with weekly updated non-redundant, cleaned and partially-predicted protein structure data [2]. The automatically updated and cleaned database is a valuable addition to PDB. As genomics studies now also incorporate “3-D genomic” structure that would be important for studying intrinsic disordered proteins, genome-scale protein folding and interactions, as well as genomic functional analysis with protein structural information, spatial gene regulation, pathway and drug target identification utilizing genomic and “3-D” protein structural information such as chromosomal contact and interaction data generated by genome conformation capturing techniques and next-generation DNA and RNA sequencing technologies, the research and database in the paper can certainly help researchers in protein science and further studies of prospective “3-D” genomics.

Green tea has been used for cancer prevention, but the mechanism is not well known. Deng and Wang’s laboratories and collaborators demonstrated that EGCG (epigallocatechin gallate) which is mostly enriched in green tea could suppress the cancer growth pathways such as proliferation by up-regulating certain miRNAs [3]. The investigation was reported as the first to obtain the miRNA and mRNA profiles from inbred mice with EGCG supplement. The authors studied mRNA expression with miRNA correlation, and presented comprehensive investigation regarding how miRNAs regulated differential expression of genes. Then the authors performed computational and statistical analysis of significant pathways and networks resulted from the regulation of miRNAs on the pathways and networks that can block the malignant transformation of cancer development. The smart experimental design to study the efficacy of EGCG at the critical time from adenoma to adenocarcinoma progression demonstrated the effectiveness of EGCG as tumour inhibitor. The research combined miRNA, mRNA, pathway and network analyses. The comprehensive study identified the roles of EGCG that influence gene expression and pathways in preventing malignant transformation. As the research presented in the paper can generate a high momentum that goes beyond the studying subject itself, hence it can provide a generalized method toward integrative genomics analysis in other disease studies.

Wang's laboratory provides useful information for human genetic studies utilizing protein sequence information [4]. The authors showed that disease causal mutations can be analysed within protein domains. This is considered as a valuable addition to traditionally DNA sequence analysis. The novelty of the work is mainly on the protein sector analysis based on residue co-evolution for sorting mutations and then relating them to diseases. The method is promising in that it can be used to identify disease-causing genes in broad genetically related diseases. Since this method was based on the information from three dimensional protein structures, it is potentially useful to the prospectively upcoming "3-D" genomics studies in the future.

## Conclusion

Elucidating the complex interplay among genes and proteins is crucial to understand molecular mechanisms of complex diseases such as cancer, but this important task is hurdled by the lack of effective computational methods with which to interpret enormous and heterogeneous multi-layer genomic data. The investigations presented in this special *BMC Genomics* supplement provided useful computational and experimental studies from identifying biomarkers and genomic variations using streamlined intelligent approaches at systems biology level to assessing impact of genomic alterations on biological or clinical outcomes. The effective utilization of multi-layer genomic data combined with high-performance computing is a significant biomedical as well as computational science problem, hence this special *BMC Genomics* supplement provides useful investigations that include systematic integration of different genomic and protein data to offer new insights into the molecular mechanisms that can help the advancement of the upcoming systems genomics studies.

## Competing interests

The authors declare that they have no competing interests.

## Authors' contributions

XQ, KY, WT, YL, ZC, HRA, JSL, AN and JYY organized external peer-reviews, contributed to reviews and selected four high-quality research papers for the special *BMC Genomics* supplement based on peer-reviews. MQY and WY wrote the article which was read and approved by all authors. 

## Declaration

The funding for publication of the article has come from the MidSouth Bioinformatics Centre, and the Joint Bioinformatics Ph.D. Program of University of Arkansas at Little Rock and University of Arkansas for Medical Sciences with NIH/NIGMS 5P20GM10342913 and ASTA award # 15-B-23.

This article has been published as part of *BMC Genomics *Volume 15 Supplement 11, 2014: Selected articles from the 2014 International Conference on Advances in Big Data Analytics. The full contents of the supplement are available online at http://www.biomedcentral.com/bmcgenomics/supplements/15/S11.
